# Modeling the response of Japanese quail to arginine intake

**DOI:** 10.7717/peerj.14337

**Published:** 2022-12-21

**Authors:** Manoela Sousa, Michele Lima, Rita Brito Vieira, Jaqueline Pavanini, Nelson José Peruzzi, Erikson Raimundo, Daniel Santos, Edney Silva

**Affiliations:** Department of Animal Science, College of Agriculture and Veterinary Sciences, Universidade Estadual Paulista, Jaboticabal, São Paulo, Brazil

**Keywords:** Arginine, Japanese quail, Modeling, Requirement, Maintenance

## Abstract

**Background:**

Understanding how Japanese quails respond to arginine intake has been an objective that previous studies have not fulfilled. The main responses to be quantified include the arginine requirement for maintenance (mg/kg^0.67^) and egg mass production (mg/g). Quantifying maintenance and production relationships are essential steps for predicting animal response. The current study aimed to describe how quails respond to arginine intake and determine arginine requirements for maintenance and egg production in Japanese quails.

**Methods:**

The experiment was carried out in a completely randomized design, with seven treatments and seven replicates with individual birds as experimental units. The arginine levels were: 2.43, 3.64, 4.85, 6.07, 9.07, 12.13, and 14.56 g/kg. The experiment lasted for eight weeks. The variables analyzed were daily arginine intake, daily arginine deposition in eggs, and body weight. The data were analyzed using a mixed model, with the experimental unit being the random effect and the experimental levels of arginine as a fixed effect. When the effect of arginine levels was detected (*P* ≤ 0.05), saturation kinetics and an exponential model with four parameters (monomolecular) were adopted. ANOVA results indicated that dietary arginine levels significantly affected (*P* < 0.01) the analyzed variables. The formulation strategy of the experimental diets allowed amplitude in the dietary arginine levels, and according to bird responses, arginine was the limiting nutrient.

**Results:**

The arginine requirement for body weight maintenance (BW^0.67^) was estimated to be 90 mg/kg BW^0.67^ by the monomolecular function. The requirement for egg mass (EM) production was estimated to be 25 mg/g per egg. A factorial model was parameterized as follows: daily arginine intake mg/bird = 90 × BW^0.67^ + 25 × EM ± 12 mg. The model was applied to data obtained from literature, and the resultant error was within the expected limit of 12 mg. The recommended daily arginine intake for the daily production of 11 g of egg and 180 g of BW was determined to be 304 mg/bird. The current study provides procedures that researchers can easily adopt.

## Introduction

The absence of pyrroline-5-carboxylate (P5C) in bird enterocytes (Flynn & Wu, 1996; Wu & Morris Jr, 1998; Wu et al., 2009) makes citrulline synthesis impossible, making arginine essential for birds (Fernandes & Murakami, 2010; Silva et al., 2012b). Understanding how Japanese quails respond to arginine intake has been an objective yet to be achieved in the production of Japanese quail eggs. Previous studies have reported that arginine does not affect the production of Japanese quail eggs (Reis et al., 2012; Cavalcante, 2013; Santos, 2013; Bulbul, Ulutas & Evcimen, 2015; Maurício et al., 2016; Maurício et al., 2018; Tuesta et al., 2018).

The lowest level of arginine tested was 0.945% by Santos (2013), and a hypothesis supported by their research was that the degree of arginine limitation used in studies carried out using the supplementation technique to establish arginine levels in the diet (Reis et al., 2012; Cavalcante, 2013; Santos, 2013; Bulbul, Ulutas & Evcimen, 2015; Maurício et al., 2016; Maurício et al., 2018; Tuesta et al., 2018), was not enough to cause limitations in protein synthesis, especially for laying birds, which mobilize body reserves to maintain egg production (Lima et al., 2018; Lima et al., 2020).

To interpret animal response, it is necessary to cause amino acid limitation; recent study carried out with broiler breeders (Lima et al., 2020) using the dilution technique to establish arginine levels, showed that birds modified egg production responses.

With the establishment of the response curve, it is possible to parameterize a factorial model to calculate arginine intake based on requirements to maintain the bird’s body weight and for egg mass production (Sakomura et al., 2015; Silva et al., 2015; Silva et al., 2019; Sarcinelli et al., 2020).

Despite its importance, information on arginine requirements for egg mass production and maintenance has not been found in literature to date. Based on the above, this research was proposed to describe how quails respond to arginine intake and to determine arginine requirements for maintenance and egg production in Japanese quails.

## Materials & Methods

The present study was conducted using Japanese quails. The Animal Ethics and Welfare Committee of Universidade Estadual Paulista (UNESP) approved all experimental procedures used in this study under protocol number 012203/17.

### Animals, housing, and experimental design

A total of 49 VICAMI® Japanese quails (Coturnix japonica) at 22 weeks were selected according to the body weight and egg production. The birds with 171 ± 2 g of body weight and 97.4 ± 1% egg production were used in this study. A 16-h photoperiod was used, with water and feed provided ad libitum. The birds were housed in an open shed with cages identified with different colors according to the respective treatment (1,200 cm^2^, nipple drinkers, and trough feeders), which comprised the study’s experimental units. The design used was completely randomized, with seven treatments and seven replicates of one bird each. In the end of the trial the animals remained in the university’s herd for egg production.

### Experimental diets

The treatments consisted of seven increasing arginine levels obtained by the dietary supplementation technique (Table 1). A basal diet was formulated to meet the nutritional requirements for metabolizable energy, minerals, vitamins, protein, and essential amino acids, except for arginine, which at 2.43 g/kg in the diet was the first arginine treatment D1.

**Table 1:** Composition (g/kg) of experimental diets.

Ingredients	D1	D2	D3	D4	D5	D6	D7
Grain Corn	607.500	614.261	621.038	627.625	627.625	636.645	647.365
Soybean Meal 46	0.000	34.032	68.062	102.402	155.566	170.000	195.852
Soy oil	21.372	21.930	22.483	23.122	29.005	29.005	29.005
Dicalcium phosphate	15.489	15.118	14.746	14.372	13.826	13.649	13.348
Limestone	69.187	69.118	69.049	68.979	68.854	68.835	68.791
Salt	3.751	3.732	3.714	3.695	3.668	3.658	3.643
Potassium chloride	7.755	6.523	5.291	4.049	2.189	1.629	0.658
DL-Methionine 98	6.513	6.055	5.596	5.134	4.457	4.240	3.871
L-Lysine 78	11.388	10.125	8.862	7.589	5.651	5.095	4.118
L-Threonine 98	4.600	3.959	3.318	2.671	1.701	1.411	0.907
L-Tryptophan 98	1.854	1.633	1.412	1.189	0.851	0.753	0.582
L-Arginine 98	0.000	0.000	0.000	0.000	1.818	3.749	5.268
L-Valine 98	5.446	4.650	3.854	3.051	1.852	1.488	0.860
L-Isoleucine 98	5.081	4.327	3.572	2.811	1.664	1.326	0.735
L-Leucine 98	9.547	8.247	6.948	5.639	3.722	3.105	2.059
L-Glycine 98	8.240	6.884	5.528	4.161	2.121	1.500	0.429
L-Phenylalanine	11.393	10.060	8.726	7.383	5.364	4.761	3.714
L-Histidine 98	2.917	2.481	2.044	1.604	0.950	0.749	0.403
L-Glutamic 98	109.364	93.467	77.569	61.548	31.455	17.675	0.000
Choline Chloride 60%	2.519	2.308	2.097	1.884	1.570	1.471	1.303
Inert	95.082	80.090	65.090	50.090	35.090	28.256	16.090
Vitamin and trace Premix^a^	1.000	1.000	1.000	1.000	1.000	1.000	1.000
Total	1000.0	1000.0	1000.0	1000.0	1000.0	1000.0	1000.0

**Notes.**

a Content (kg/diet), vit A, 7.000 IU; vit D3, 2.000 IU; vit E, 8 IU; vit K3, 2 mg; vit B1, 1 mg, vit B2, 3.5 mg; vit B6, 2 mg; vit B12, 5 mcg/kg; niacin, 25 mg; chlorine, 0.26 g; pantothenate acid, 10 mg; copper, 8 mg/kg; iron, 50 g; manganese, 70 g; zinc, 50 g; iodine, 1.2 mg and selenium 0.2 mg.

The other treatments were formulated with increasing arginine levels as follows: D2, 3.64 g/kg; D3, 4.85 g/kg; D4, 6.07 g/kg; D5, 9.70 g/kg; D6, 12.13 g/kg; and D7, 14.56 g/kg, resulting in an amplitude of 600% ([14.56/2.45] × 100) as shown on Table 2. In addition to corn and soybean meal, industrial amino acids were used to balance the essential amino acid and crude protein levels in the diets. Recommendations for nutritional requirements were obtained from Rostagno et al. (2011), as shown in Table 2.

**Table 2:** Nutritional levels of experimental diets.

Nutrients	D1	D2	D3	D4	D5	D6	D7
ME_*n*_^a^, MJ/kg	12.55	12.55	12.55	12.55	12.55	12.55	12.55
Crude protein, g/kg	170.00	170.00	170.00	170.00	170.00	170.00	170.00
Lysine, g/kg	10.45	10.45	10.45	10.45	10.45	10.45	10.45
Methionine + Cystine, g/kg	8.57	8.57	8.57	8.57	8.57	8.57	8.57
Threonine, g/kg	6.27	6.27	6.27	6.27	6.27	6.27	6.27
Tryptophan, g/kg	2.20	2.20	2.20	2.20	2.20	2.20	2.20
Glycine + Serine, g/kg	12.51	12.51	12.51	12.51	12.51	12.51	12.51
Valine, g/kg	7.84	7.84	7.84	7.84	7.84	7.84	7.84
Isoleucine, g/kg	6.79	6.79	6.79	6.79	6.79	6.79	6.79
Leucine, g/kg	15.68	15.68	15.68	15.68	15.68	15.68	15.68
Histidine, g/kg	4.39	4.39	4.39	4.39	4.39	4.39	4.39
Phenylalanine + Tyrosine, g/kg	14.81	14.81	14.81	14.81	14.81	14.81	14.81
Potassium, g/kg	6.00	6.00	6.00	6.00	6.00	6.00	6.00
Sodium, g/kg	1.55	1.55	1.55	1.55	1.55	1.55	1.55
Calcium, g/kg	30.00	30.00	30.00	30.00	30.00	30.00	30.00
Non-phytate phosphorus, g/kg	3.23	3.23	3.23	3.23	3.23	3.23	3.23
Choline, mg/kg	1500.00	1500.00	1500.00	1500.00	1500.00	1500.00	1500.00
Linoleic acid, g/kg	22.84	23.50	24.14	24.84	29.85	28.92	28.93
Crude fiber, g/kg	10.94	12.93	14.92	16.93	22.68	23.00	22.42
Arginine, g/kg	2.45	3.64	4.85	6.07	9.7	12.13	14.56
Percentage in relation to BTPS^b,c^, %	20	30	40	50	80	100	120

**Notes.**

a ME_*n*_, Metabolizable energy corrected for nitrogen balance.

b Percentage obtained: [Arginine/12.13] × 100; 12.13 g/kg according to BTPS (Rostagno et al., 2011).

c BTPS, Brazilian Tables for Poultry and Swine, Rostagno et al. (2011).

The nutritional matrix was adjusted for the composition of the examined ingredients. The bromatological composition and amino acid content of corn and soybean meal were analyzed using a near-infrared spectrometer (Evonik Industries AG, Essen, Germany).

### Experimental procedures, measurements, and variables analyzed

The experiment lasted eight weeks, with data collection occurring in the last four weeks, according to Silva et al. (2019). Temperature, egg production, and mortality were measured daily. On three consecutive days each week, the eggs were weighed to obtain the average weight. Feed leftovers were evaluated biweekly. Quail weighing was performed at the beginning and end of the experiment. The mean temperature and relative humidity throughout the experimental period were 26 °C and 40%, respectively.

The variables analyzed were feed intake (FI, g/bird.d), body weight (BW, g), EP (%), egg weight (EW, g), and feed conversion ratio by egg output (FCR, g/g), corrected for mortality, body weight modification (cBW, g/bird), egg output (EO, g/bird.d), arginine intake (mg/bird.d), arginine deposition in egg (mg/bird.d), and arginine mobilization of BW (mg/bird.d). The protein and arginine composition of the egg and body of Japanese quails used to calculate arginine deposition in the egg and arginine mobilization are shown in Table 3.

**Table 3:** Protein and arginine content of whole egg and body protein.

Egg^a^		Body^b^	
Protein, g/kg	Arginine g/100 CP	Protein, g/kg	Arginine, g/kg
129.9	5.14	20.3	9.40

**Notes.**

a Ali & El-Aziz (2019).

b Sharif, Kamarudin & Huda (2019).

### Description of the response to arginine deposition in the egg, estimate of the requirement for maintenance, and total efficiency of the utilization of arginine

The variables arginine intake (*X*) and arginine deposition in eggs (*Y*) were standardized according to the unit system per kilogram of metabolic weight (BW^0.67^), converting them into mg/kg BW^0.67^, according to Silva et al. (2020). Two models were used to interpret the relationship between *Y* and *X*, a monomolecular function was modified for four parameters adapted from Kebreab et al. (2008) and the saturation kinetics model (Mercer, 1982).

The monomolecular function modified for four parameters used in the current study was as follows:

(1.0) \begin{eqnarray*}Y={R}_{\mathrm{max}}-{R}_{\mathrm{min}}[1-{e}^{(-k(X-Xm))}].\end{eqnarray*}

where *Y* is the daily arginine deposition in eggs (mg/kg BW^0.67^), *X* is the daily arginine intake (mg/kg BW^0.67^), *R*_max_ is the maximum arginine deposition response in eggs (mg/kg BW^0.67^), *R*_min_ is the minimum arginine deposition response in eggs (mg/kg BW^0.67^), *k* is the slope of the function, and *X*_*m*_ is the daily maintenance requirement (mg/kg BW^0.67^).

The daily arginine maintenance requirement (mg/bird) obtained by the monomolecular function modified for four parameters applying Eq. (1.0) modified to *R*_min_ > 0.

(1.1) \begin{eqnarray*}X{{|}}_{\mathrm{Y 0}}=B{W}^{0.67}\times [{X}_{m}].\end{eqnarray*}

The other model is saturation kinetics, according to Eq. (2.0).

(2.0) \begin{eqnarray*}Y=[{R}_{\mathrm{min}}\times {k}_{m}^{n}+{R}_{\mathrm{ max}}\times {X}^{n}]/[{k}_{m}^{n}+{X}^{n}]\pm .\end{eqnarray*}

where *Y* is the daily arginine deposition in the egg (mg/kg BW^0.67^), *X* is the daily arginine intake (mg/kg BW^0.67^), *R*_max_ is the maximum arginine deposition response in the egg (mg/kg BW^0.67^), *R*_min_ is the minimum arginine deposition response in the egg (mg/kg BW^0.67^), *k*_*m*_ is the daily arginine intake for 0.5 of (*R*_max_ + *R*_min_), and *n* is the apparent kinetic order of the response with respect to *X* as *X* approaches zero.

The daily arginine requirement for maintenance (mg/bird) obtained by the saturation kinetics model using Eq. (2.1), modified to *R*_min_ > 0, is as follows:

(2.1) \begin{eqnarray*}X{{|}}_{\mathrm{Y 0}}=B{W}^{0.67}\times [{k}_{m}[{R}_{\mathrm{min}}/{R}_{\mathrm{max}}]^{1/n}].\end{eqnarray*}

The total efficiency of utilization of arginine egg deposition was obtained by differentiation of Eqs. (1.0) and (2.0), applying d*f*/d*x*.

### Efficiency of utilization of arginine

The efficiency of arginine utilization was corrected for maintenance and mobilization. Laying hens use body reserves as an amino acid source to maintain egg production when dietary support is lacking. Therefore, the amount of arginine used for maintenance and body mobilization was subtracted from the ingested dietary amino acid. The efficiency of utilization calculated for each experimental unit was regressed as a function of arginine intake for protein deposition in the egg, using broken-line regression (Robbins, Saxton & Southern, 2006) and Eqs. (1.0) and (2.0).

### Adjustment and model selection statistics

The criteria adopted for the selected models were the determination coefficient adjusted for the number of parameters (*R*^2^_adj_ ) and the Bayesian information criterion (BIC).

### Model assessment statistics

The models were subjected to residue analysis as described by St-Pierre (2003). The residues (observed–predicted) were regressed according to the predicted values according to the following model:

(3.0) \begin{eqnarray*}{r}_{i}={b}_{0}+{b}_{1}({y}_{i}-\mathrm{\mu })+{e}_{i}.\end{eqnarray*}

where *r*_*i*_ is the residual value for all *i*th observations, *b*_0_ and *b*_1_ are the parameter estimates, *y*_*i*_ is the predicted value for all *i*th observations, µis the average value for all predicted *y* values, and *e*_*i*_ is the error of the regression of the residuals over the predicted values. The decision rule assumed that the model was impartial as correlation approaches 1 and when *R*^2^_adj_ approaches 0, *i.e.,* the residuals are not correlated with the predictions. The slope value, *b*_1_, as a function of *y*_*i*_, must tend towards zero for the model to be impartial (St-Pierre, 2003; Silva et al., 2020). Therefore, the value of *b*_1_ ≠ 0 indicates the model’s prediction bias (St-Pierre, 2003; Silva et al., 2020). The *b*_0_ value indicates the general error and is related to the scale of the difference (St-Pierre, 2003; Silva et al., 2020). The precision value of the model was calculated by considering the 1 − *R*^2^_adj_ (St-Pierre, 2003; Silva et al., 2020).

### Statistical analysis

The data were analyzed for the assumptions of homoscedasticity of variance (Brown-Forsythe) and normality of errors using PROC UNIVARIATE, then outliers were removed according to test procedures (Anderson-Darling, Shapiro–Wilk T and, Cramér-von Mises Test).

The experimental unit was considered the random effect, and the experimental arginine level was considered the fixed effect. The variables were subjected to orthogonal contrast analysis to determine the linear and quadratic effects of arginine levels. When an effect was detected, considering a significance of 0.05 (*P* ≤ 0.05), regression analysis was applied using the monomolecular function modified for four parameters, saturation kinetics, and broken-line models. Statistical analyses and estimation of model parameters were performed using SAS 9.4 (Statistical Analysis for Windows, SAS Institute Inc., Cary, NC; 2014).

## Results

### Responses to dietary arginine levels

The formulation strategy of the experimental diets allowed amplitude in the dietary arginine levels, and according to the responses of the quails, arginine was the limiting nutrient.

ANOVA indicated that the arginine levels in the diet significantly affected (*P* < 0.01) the analyzed variables (Table 4). The reduction in feed intake of D1, formulated with 2.43 g of arginine per kg, was 51.4% (13.2/25.7) in relation to the maximum value observed with the 12.13 g arginine per kg diet (D6). Birds fed the 2.43 g of arginine per kg diet (D1) reduced arginine intake by approximately 90% (100 − (32/328.7) × 100), in relation to the maximum value observed with the 14.56 g arginine per kg diet (D7).

**Table 4:** Responses for feed intake, arginine intake, egg production, egg weight, egg mass, feed efficiency, arginine deposition in egg, body weight, change body weight, and arginine mobilization (mg/bird).

Diets	Level	Feed intake	Arginne intake	Egg production	Egg weight	Egg mass	Feed efficiency	Arginine deposition in egg	Body weight	Change body weight	Arginine mobilization
	g/kg	g/bird d	mg/bird d	%/bird d	g	g/bird d	g/g	mg/bird d	g	g/bird	mg/bird d
D1	2.43	13.2	32.0	13.3	5.4	1.0	0.08	6.8	147.2	−34.8	−11.9
D2	3.64	15.5	56.3	36.3	8.8	3.2	0.21	22.4	149.4	−34.3	−11.7
D3	4.85	17.1	82.9	51.2	8.9	4.6	0.27	32.2	151.3	−22.1	−7.5
D4	6.07	19.3	116.9	80.0	9.5	7.6	0.39	53.2	167.1	−24.6	−7.8
D5	9.07	23.4	212.6	92.1	10.8	10.0	0.43	70.2	174.2	−8.9	−3.0
D6	12.13	25.7	311.3	96.4	10.9	10.5	0.41	73.6	179.7	−0.8	−0.3
D7	14.56	22.6	328.7	95.8	10.2	9.8	0.43	68.6	173.3	5.0	1.7
Average		19.53	162.98	66.45	9.20	6.64	0.32	46.71	161.8	−17.0	−5.8
SEM		0.78	19.80	5.77	0.44	0.64	0.02	4.52	2.8	2.9	1.0
*P*-value											
Anova		<0.0001	<0.0001	<0.0001	0.0007	<0.0001	<0.0001	<0.0001	0.0043	<0.0001	<0.0001
Linear effect		<0.0001	<0.0001	<0.0001	0.0001	<0.0001	<0.0001	<0.0001	0.0001	<0.0001	<0.0001
Quadratic effect		0.0095	<0.0001	<0.0001	0.0183	<0.0001	<0.0001	<0.0001	0.1894	0.4421	0.4421

The dietary limitation of arginine at 2.43 g/kg in D1, affected the egg production (14% = 13.3/96.4 × 100; *P* < 0.001), and egg weight (50% = 5.4/10.9 × 100; *P* < 0.001), in relation to the maximum value observed with the 12.13 g arginine per kg diet (D6). A greater limitation was observed for egg production, which reduced proportionally, three times more than egg weight (3.6 = 50/14) (Table 4). Birds fed lowest level (2.43 g/kg, D1) produced egg mass responses close to zero. The maximum response was 10.5 g/bird d for an arginine level of 12.13 g arginine per kg of diet (D6). The maximum feed efficiency was 0.43 or 43% of feed conversion to product, *i.e.,* egg mass (Table 4).

For a diet with a lower arginine level (2.43 g/kg, D1), a 32 mg/day supply was insufficient to maintain the deposition of 6.8 mg of arginine in the egg per day (Table 4) and meet the demand for basal metabolism. The birds in this treatment mobilized 12 mg of arginine per day to maintain an approximate production of one egg per week.

The increase in arginine concentration in the diet increased arginine deposition in the egg and reduced the body’s mobilization to the level of the 12.13 g/kg diet (D6) compared to lower arginine level (2.43 g/kg, D1). In D7, there was no increase in arginine deposition in the egg, but there was an increase in arginine deposition in the body compared compared to arginine level (12.13 g/kg, D6) (Table 4).

### Maximum and minimum responses to arginine deposition in the egg, maintenance requirement, and total efficiency of utilization of arginine

Linear and quadratic effects were verified using orthogonal contrasts (*P* < 0.001). Arginine levels were considered as a fixed effect, and the experimental unit was considered a random effect. The monomolecular function with four parameters and saturation kinetics were fitted using a non-linear mixed model procedure, considering the birds’ maximum response as a random effect.

The relationship between arginine deposition in the egg and arginine intake was adjusted with R^2^ above 90% in both models (Table 5). The BIC values obtained were 329 for the monomolecular function with four parameters and 327 for saturation kinetics (Table 5).

**Table 5:** Parameters estimated and statistical for fit and assessment of the models monomolecular with four-parameters (M1) and saturation kinetics (M2) to predict arginine deposition in quail eggs.

Models		R^2^Adj^a^	BIC^b^	*b*_0_ ^c^	*b*_1_ ^d^	Precision^e^
M1	*Y* = 249 − 11[1 − *e*(−0.003934(*X* − 90))]	0.93	329	−0.07	0.006	0.999
M2	*Y* = [21 × 291^3.413^ + 232 × *X*^3.413^]/[291^3.413^ + *X*^3.413^]	0.91	327	−4.79	−0.054	0.950

**Notes.**

a R^2^Adj, coefficient of determination adjusted for number of parameters.

b BIC, Bayesian Information criterion.

c *b*_0_, difference range.

d *b*_1_, prediction bias.

e Precision, 1-R^2^.

The monomolecular function with four parameters:

\begin{eqnarray*}Y=250(\pm 5)\times -11(\pm 5)(1-{e}^{[-0.003934(\pm 0.00043)\times [X-90(\pm 12)]]}). \end{eqnarray*}

Saturation kinetics:

\begin{eqnarray*}Y& =& [21(\pm 15)\times 291(\pm 15)^{3.413(\pm 0.606)}+232(\pm 13)\times {X}^{3.413(\pm 0.606)}]/[291(\pm 15)^{3.413(\pm 0.606)}\nonumber\\\displaystyle & & +~{X}^{3.413(\pm 0.606)}] \end{eqnarray*}

where *Y* is the daily arginine deposition in eggs (mg/kg BW^0.67^), and *X* is the daily arginine intake (mg/kg BW^0.67^). The error associated with each parameter is important in supporting the inference of the biological interpretation of the model’s parameters on the animal response. The errors for the monomolecular function with four parameters were 2, 5, 11, and 13% for *R*_max_, *R*_min_, *k*, and *X*_*m*_, respectively. The saturation kinetics model errors were 6, 71, 18, and 5% for *R*_max_, *R*_min_, *n*, and *k*_*m*_, respectively.

**Figure 1:**
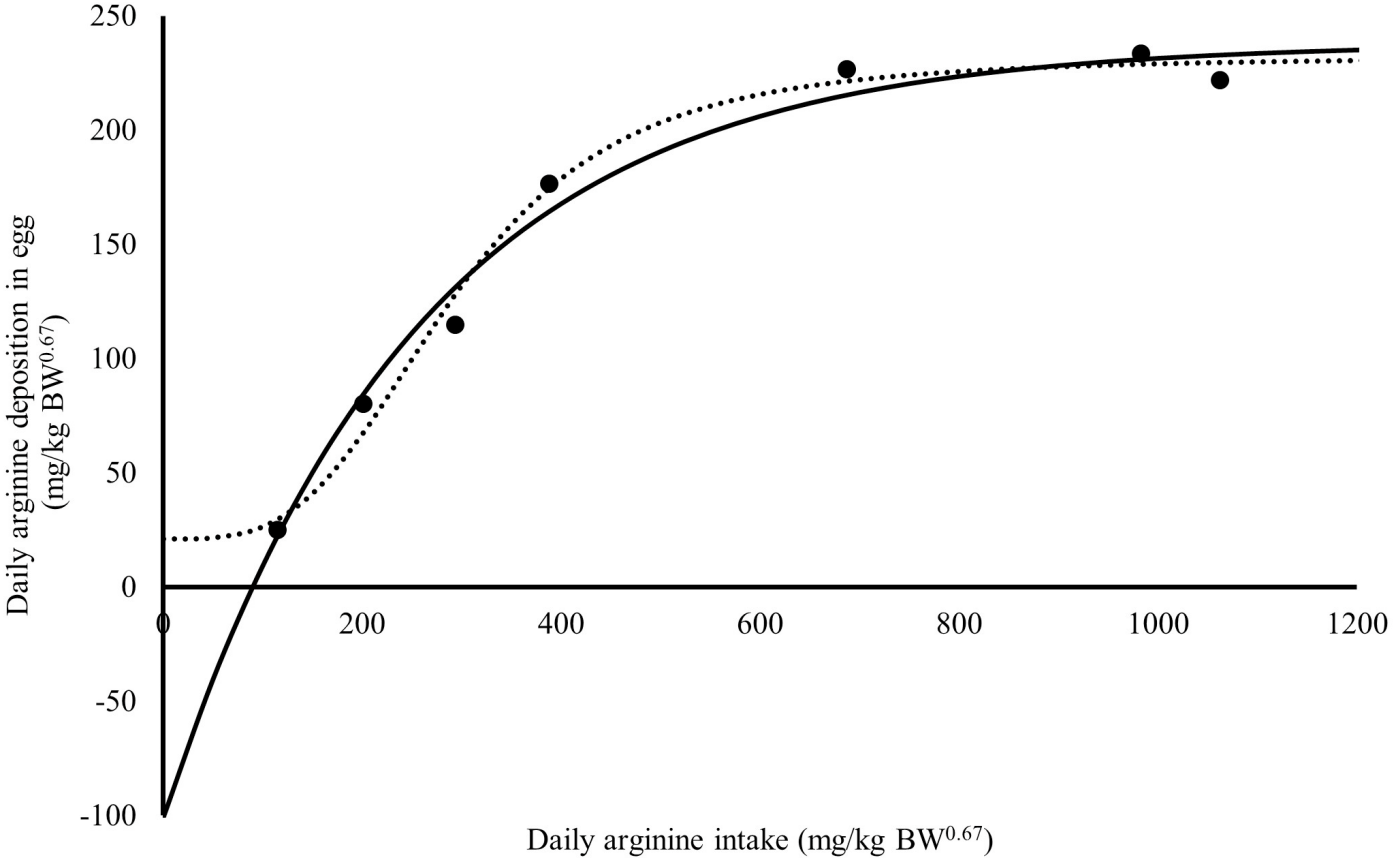
Relation between daily arginine deposition in egg (mg/kg BW^0.67^), and daily arginine intake (mg/kg BW^0.67^). A dot indicates the observed values daily arginine deposition in egg; the continuous line indicates the predicted values daily arginine deposition in egg by Monomolecular four-parameters; the dotted line indicates the predicted values daily arginine deposition in egg by Kinetic Saturation.

**Figure 2:**
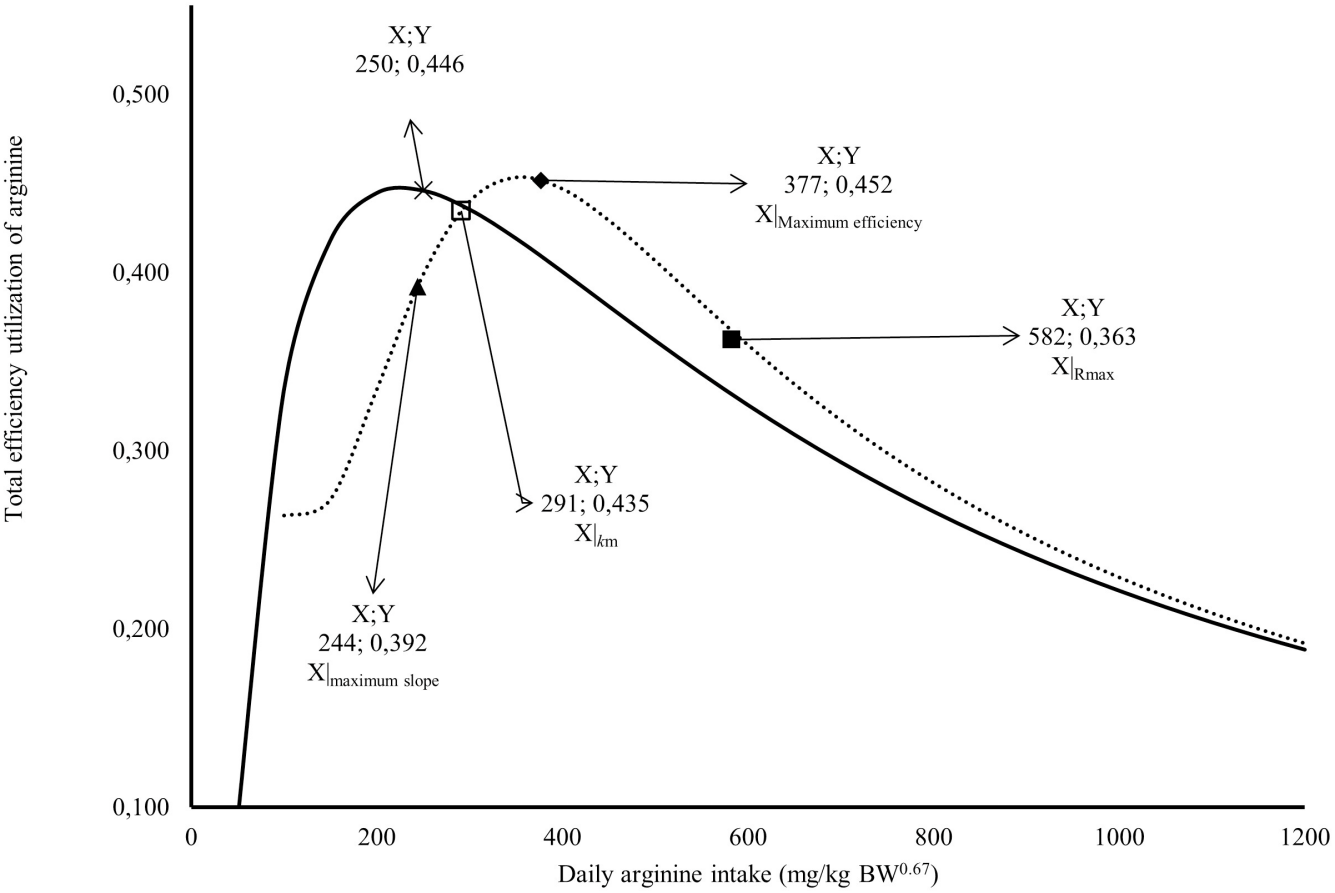
Relation between total efficiency utilization of arginine deposited in egg, and daily arginine intake (mg/kg BW^0.67^). A full square indicates arginine intake at maximum response; An empty square indicates arginine intake at maximum efficiency; a lozenge indicates arginine intake for intake for 1/2 (*R*_max_ + *R*_min_); a triangle indicates arginine intake at the maximum slope; × Arginine intake at maximum efficiency; the continuous line indicates the predicted values total efficiency utilization of arginine deposited in egg by Monomolecular four-parameters; the dotted line indicates the predicted values total efficiency utilization of arginine deposited in egg by Kinetic Saturation.

The *R*_max_ value estimated for Japanese quail was 249 mg daily of arginine deposition in eggs per kg BW^0.67^ (Fig. 1). This value was similar between both models when considering the error (245 = 232 + 13). The *R*_min_ value estimated for Japanese quail was 11 mg daily arginine deposition in eggs per kg BW^0.67^, based on the monomolecular function with four parameters (Fig. 1). Even considering the error, *R*_min_ was different between for the models. This difference is associated with the shape of the *R*_min_ curve, especially in the saturation kinetics model (Fig. 1). The *k* (0.003934) and *n* (3.413) parameters of the monomolecular function with four parameters and saturation kinetics model, respectively, are related to the response rate per unit of ingested arginine (Fig. 1). The saturation kinetics function had an inflection point (Fig. 2). The parameter *k*_*m*_ is related to intake in obtaining half the maximum response (*R*_max_ + *R*_min_) of the arginine deposition response in the egg and was estimated to be 291 mg/kg BW^0.67^. The other two pieces of information near the *k*_*m*_ aid in interpreting quail response to arginine intake (Fig. 2).

### Efficiency of utilization and requirement of arginine for egg production

According to monomolecular function with four parameters, the maintenance was estimated at 90 mg/kg BW^0.67^, thus, the efficiency of arginine utilization was calculated by correcting the contribution of maintenance and mobilization of arginine to support egg production. The efficiency was obtained from the relationship between arginine deposition in eggs and arginine intake. The total efficiency of utilization of arginine (*k*_total_) obtained using the broken line-model (*Y* = 225(±6) − 0.53(±0.04) × [484(±22) − *X*], *R*^2^ = 0.93, *k*_total_: 225/484 = 0.46 or 46%) was the 0.46 or 46%.

The model proposed in this research was evaluated using information from three most recently published articles (Reis et al., 2012; Maurício et al., 2016; Tuesta et al., 2018) on the arginine requirement for Japanese quails (Table 6).

**Table 6:** Results of application of the proposed model in data published in the literature.

Inputs of the factorial model^a^	Reis et al. (2012)			Maurício et al. (2016)			Tuesta et al. (2018)
Body weight, kg	0.170^b^	0.180^b^	0.190^b^	0.170^b^	0.180^b^	0.190	0.181
Daily egg mass, g/bird	10.72	10.72	10.72	11.13	11.13	11.13	9.75
Daily Arginine intake observed	289	289	289	302	302	302	259
Predicted by model	295	297	298	306	307	308	272.4
Error (observed-predict)	−7	−8	−9	−4	−5	−6	−13.4

**Notes.**

a The model predicted daily arginine intake mg/bird = 90 × BW^0.67^ + 25 × EM, BW is Body weight; EM is daily egg mass.

b Value considers this research for simulation (Reis et al., 2012; Maurício et al., 2016) did not report the body weight of the quails.

## Discussion

### Responses to dietary arginine levels

The method applied made it possible to obtain egg mass responses close to zero for the treatment with lower arginine level in the diet (2.43 g/kg, D1). The maximum response obtained was 10.5 g/bird d for an arginine level of 12.13 g arginine per kg of diet (D6) (Table 4). Thus, it was possible to describe the productive responses close to the bird’s maintenance up to the stability region, related to the level of 14.56 g of arginine per kg of diet (D7), which was not limited to improving quail responses.

The value of feed efficiency (0.43 or 43% of feed conversion to product) suggested that a smaller dose, 9.07 g/kg (D5), was sufficient to achieve the optimal result for greater feeding efficiency. Although the level of 14.56 g/kg of arginine provided the same feed efficiency, this was attributed to a reduction in feed intake since there was no increase in egg mass production. The birds fed with a lower arginine level (2.43 g/kg, D1) mobilized 12 mg of arginine per day to maintain an approximate production of one egg per week. This result shows that reproduction is a priority physiological phenomenon in Japanese quails.

### Maximum and minimum responses to arginine deposition in the egg, maintenance requirement, and total efficiency of utilization of arginine

According to the results obtained, arginine deposition in the egg represents a priority physiological phenomenon for Japanese quails. For this reason, arginine deposition in the egg was selected for interpretation using a monomolecular function with four parameters and saturation kinetics.

According to the rule, the lowest BIC value should be used to select the model that best fits the analyzed data; however, the difference between the two units was too small to decide. To aid in this interpretation, we analyzed the prediction residues for each model. According to the results for *b*_0_ (scalar difference), *b*_1_ (prediction bias), and precision (Table 5), the monomolecular function with four parameters showed the smallest scalar difference, smallest bias, and highest precision in predicting the observed values.

The parameter *X*_*m*_ is related to arginine intake for maintenance and was estimated to be 90 ±12 mg/kg BW^0.67^ by the monomolecular function with four parameters (Fig. 1). Using the saturation kinetics model parameters, it was possible to obtain a value of 144 mg/kg BW^0.67^, analogous to *X*_*m*_(*X*|Y 0 = *k*_*m*_[*R*_min_/*R*_max_]^1/*n*^); however, there is no information on the error of this calculation. Therefore, precision depends on obtaining negative responses on the ordinate axis (Mercer, 1982).

The first is the intake at the maximum slope calculated at 244 mg/kg BW^0.67^, and the second is the intake at maximum efficiency calculated at 377 mg/kg BW^0.67^ (Fig. 2). The intake at the maximum slope provides one area with greater sensitivity in obtaining responses to changes in arginine intake. The intake at maximum efficiency provides the arginine intake where the response was maximum for minimum arginine intake. The monomolecular function with four parameters does not have an inflection point, which is a limiting factor for interpreting the responses. However, this is a characteristic of all exponential models. In Fig. 2, a point was calculated in the area of maximum efficiency of the utilization value. In both models, similar values (0.45 or 45%) were obtained for the total efficiency of arginine utilization.

### Efficiency of utilization and requirement of arginine for egg production

When applied correcting for the maintenance contribution, the value of efficiency of utilization of arginine (*k*_Intake-Maintenance_) was 0.57 or 57%, according to the broken-line model (*Y* = 225 (±6) −0.53 (±0.04) × (394(±22) − *X*), *R*^2^ 0.93, *k*_Intake-Maintenance_: 225/394 = 0.57 or 57%). With a correction for maintenance plus mobilization, efficiency of arginine utilization was 0.59 or 59%, based on the broken line-model (*Y* = 225 (±6)−0.49 (±0.04) × (380(±24) −*X*), *R*^2^ 0.93, *k*_Intake−[Maintenance + mobilization]_: 225/380 = 0.59 or 59%).

Based on the deposition value of arginine in the egg of 7.3 mg of arginine per g of egg and the values of efficiency of utilization of 0.46, 0.57, and 0.59, the values of the requirements for egg mass production (*R*_eq_) were calculated by dividing the deposition by each value of efficiency to obtain 15.3, 12.3, and 11.9 mg of arginine per g egg mass, respectively. The broken-line model approach commonly yields lower requirements than non-linear models (Fatufe & Rodehutscord, 2005). All values obtained for *R*_eq_ were based on area, limiting the use of the broken-line model to define the breakpoint, and according to Silva et al. (2020), this point is similar between linear and non-linear models.

In contrast, previous studies have not described limiting arginine intake (Reis et al., 2012; Cavalcante, 2013; Santos, 2013; Bulbul, Ulutas & Evcimen, 2015; Maurício et al., 2016; Maurício et al., 2018; Tuesta et al., 2018). The current study showed that Japanese quails respond to arginine intake. The diets formulated based on corn, soybean meal, and industrial amino acids (Table 1) allowed us to obtain large-amplitude levels in the diet, and the results support that the method applied was adequate for describing the response of 1 to 10.5 g of egg mass daily per bird (Table 4).

Comparisons with other studies demonstrate the extent to which requirement estimates may vary when they are based on individual studies with particular designs, genotypes, feeds, and environments (Fatufe & Rodehutscord, 2005; Kebreab et al., 2008) modeling requirements is a viable alternative (Fatufe & Rodehutscord, 2005; Kebreab et al., 2008; Silva et al., 2019; Silva et al., 2020).

The curve-response established in the present study allowed us to obtain the maintenance coefficient and requirement for Japanese quail egg mass (EM). A factorial model was parametrized as follows: daily arginine intake (DAI) mg/bird = 90 × BW^0.67^ + 12 × EM, for EM ≤ 11 g/bird. Owing to the linear relationship of the parameters, a plateau should be considered to avoid overestimation of arginine intake. The value calculated to DAI was 160 ± 19 mg/bird (BW = 0.180 kg), considering 11 g/bird of EM. Large errors were observed in the treatments close to the decision-making area, underestimating arginine intake.

However, this value is much lower than the measured values of treatments D6, D5, and D4, as shown in Table 4. To reproduce the measured values, only the maintenance was subtracted from the total intake and divided by the deposition in each treatment, according to the following equation: *R*_eq_ mg/g = [arginine intake − maintenance]/egg mass. The values obtained for each treatment were 7, 10, 13, 12, 19, 27, and 31 mg of arginine per g of egg mass for D1, D2, D3, D4, D5, D6, and D7, respectively. Using the broken-line model between egg production (%) and arginine requirement (*R*_eq_ mg/g), we found a requirement value for egg production (EP), estimated at 25 mg of arginine per g of egg mass, according to the model: EP = 93 − 3.1 × (25 − *R*_eq_), where (25 − *R*_eq_) is defined as zero when *R*_eq_ > 25. Using 25 mg/g (DAI mg/bird = 90 × BW^0.67^ + 25 × EM), the value calculated to meet DAI was 304 ±12 mg/bird (BW = 0.180 kg) considering 11 g/bird of EM.

All analyses, regardless of the mathematical model, suggest that the maximum utilization efficiency of daily intake was 377-484 mg/kgBW^0.67^ or 119–153 mg/bird. However, when parameters were extracted from this area of the curve-response (119–153) to predict the animal response, the distance between observations and predictions was not acceptable, especially close to the decision-making region.

A hypothesis to justify our observations may be associated with the high digestibility of the experimental diets formulated with limiting arginine levels, D1, D2, and D3. These diets contained an expressive contribution of free amino acids in the purified form, and at increased arginine levels, free amino acid supplementation was reduced with increased soybean meal inclusion. This methodological characteristic may have influenced the high efficiency in the limiting region, generating parameters that estimated arginine intake lower than the values determined in this test. The model (DAI mg/bird = 90 × BW^0.67^ + 25 × EM) considering the requirement of 25 mg of arginine per g of egg mass is preferable because it predicts values close to those obtained in the decision-making region.

According to information from three most recently published articles (Reis et al., 2012; Maurício et al., 2016; Tuesta et al., 2018) on the arginine requirement for Japanese quails, the present model could predict arginine intake with an acceptable error within the established ±12 mg of arginine. The errors obtained by applying this model using data from Reis et al. (2012) ranged from −7 to −9 mg. Using data from Maurício et al. (2016), the errors were smaller than expected, ranging from −4 to −6 mg. With data obtained from Tuesta et al. (2018), the error obtained was −13 mg, closer to the expected limit (Table 6).

The proposed model was applied to the data of Allen & Young (1980), who pioneered amino acid nutrition for Japanese quails. The predicted arginine intake was 208 mg/bird, considering 134 g of body weight, 7.37 g/day of egg mass, differing by −5 mg from the arginine intake recommended by Allen & Young (1980).

In general, the model suggests a lower intake than the values reported in previous studies (Allen & Young, 1980; Reis et al., 2012; Maurício et al., 2016; Tuesta et al., 2018). However, it is not possible to declare that the model underestimates arginine intake since none of the aforementioned studies described dietary arginine limitations. Therefore, for this simulation, the treatment information from each study with a lower arginine concentration in the diet was considered.

The results of Bulbul, Ulutas & Evcimen (2015) were not used in this simulation because of the high levels tested. The minimum daily intake of arginine (342 mg/bird) was greater than the maximum level tested in the current study (328 mg/bird). Bulbul, Ulutas & Evcimen (2015) found no effect on the productive responses of birds and reported that excess arginine impaired eggshell quality.

The consolidated information on poultry nutrition provides the ideal relationship between amino acids and lysine. Due to the lack of quail responses to dietary levels of arginine in the diet, some researchers (Silva et al., 2012a) have maintained the recommendation of the NRC (NRC, 1994), which suggests an Arg:Lys ratio of 126%.

Using the factorial model by Silva et al. (2019), which considers the requirement of maintaining lysine (156 × 0.180^0.75^ or 136 × 0.180^0.67^) and egg mass production (21 × 11), the daily lysine intake obtained (274 mg/bird) results in a ratio of 1.08 or 108% (Arg/Lys: 304/274 = 1.08). The ratio arginine:lysine to produce 1 g of egg mass was 1.14 or 114% considering 25 mg/g determined in the current study and 21 mg of lysine found by Silva et al. (2019). This ratio was close to the values of 116 and 115% reported by Rostagno et al. (2011); Rostagno et al. (2017), respectively. However, the information provided in this research was extracted from a response curve derived from dietary arginine limitations.

## Conclusions

The formulation strategy of the experimental diets allowed amplitude in the dietary levels of arginine, and according to the responses of the birds, arginine was the limiting nutrient. The arginine requirement for maintenance was estimated to be 90 mg/kg^0.67^ of metabolic body weight. The egg mass production requirement was estimated to be 25 mg/g per egg. The recommended daily arginine intake to produce 11 g of egg and 180 g of body weight was calculated to be 304 mg/bird.

##  Supplemental Information

10.7717/peerj.14337/supp-1 File S1 Variables collected This dataset contain the variables collected during the trial. The columns are: the observation number, cage, treatment, replicate, level of arginine, feed intake, egg production, egg weight, initial body weight and final body weight, respectively.

10.7717/peerj.14337/supp-2 File S2 Procedures for statistical analysis The procedures correspond to the step by step used in the SAS software to obtain the parameters used in the mathematical models for manuscript.

10.7717/peerj.14337/supp-3 Supplemental Information 1 Author Checklist

## Additional Information and Declarations

### Data Availability

The following information was supplied regarding data availability: The data are available in the Supplemental Files.
