# Supplementary material for: Modeling the response of Japanese quail to arginine intake
Source: PeerJ. 2022 Dec 21;10:e14337. doi: 10.7717/peerj.14337 (PMC9789692; doi:10.7717/peerj.14337)
Supplement: File S2 — The procedures correspond to the step by step used in the SAS software to obtain the parameters used in the mathematical models for manuscript. [file peerj-10-14337-s002.docx]

**Modeling Japanese quail egg production responses to arginine intake by different mathematical functions: A factorial approach**

The procedures declared below correspond to the step by step used in the SAS software to obtain the parameters used in the mathematical models for manuscript.

The data were analyzed for assumptions of homoscedasticity of variance (Brown-Forsythe) and normality of errors using PROC UNIVARIATE, then outliers were removed according to test procedures (Anderson-Darling, Shapiro-Wilk T and, Cramér -von Mises Test). Considering this information, each treatment had 7, 6, 3, 5, 5, 3 and 6 repetitions, for treatments 1, 2, 3, 4, 5, 6 and 7, respectively.

**data** ReportManoela;

**input** Obs Cage Treatments$ RP Level FeedIntake EggProduction EggWeight InitialBW FinalBW;

**cards**;

1 81 T10 1 0.243 12.525 10.714 9.150 143.850 117.860

2 90 T10 2 0.243 11.476 28.571 5.882 149.920 113.840

3 96 T10 3 0.243 13.686 0.000 0.000 174.090 131.720

4 106 T10 4 0.243 10.539 14.286 6.985 157.140 110.990

5 112 T10 5 0.243 14.202 25.000 7.712 178.920 145.240

6 122 T10 6 0.243 15.025 0.000 0.000 173.050 146.510

7 127 T10 7 0.243 14.710 14.286 8.077 175.220 142.270

8 86 T11 1 0.364 15.932 42.857 7.553 154.730 126.800

9 92 T11 2 0.364 12.797 32.143 7.978 181.830 129.540

10 100 T11 3 0.364 14.529 21.429 8.900 155.030 127.720

11 105 T11 4 0.364 18.731 28.571 9.870 166.900 133.410

12 111 T11 5 0.364 15.068 46.429 8.561 179.260 144.470

13 126 T11 7 0.364 15.798 46.429 9.870 161.440 131.280

14 84 T12 1 0.485 17.482 57.143 8.117 173.070 138.720

15 88 T12 2 0.485 15.942 39.286 8.800 165.510 140.050

16 107 T12 4 0.485 17.884 57.143 9.881 148.490 141.870

17 82 T13 1 0.607 21.233 82.143 9.698 169.180 148.360

18 101 T13 3 0.607 18.100 78.571 8.345 180.300 146.620

19 108 T13 4 0.607 19.430 78.571 9.371 175.030 152.200

20 110 T13 5 0.607 19.581 92.857 9.744 196.810 172.630

21 119 T13 6 0.607 17.946 67.857 10.173 171.750 150.020

22 85 T14 1 0.907 25.756 100.00011.700 197.970 190.750

23 89 T14 2 0.907 21.800 82.143 10.199 177.620 178.770

24 95 T14 3 0.907 24.511 89.286 10.327 180.610 166.790

25 102 T14 4 0.907 23.411 96.429 10.032 156.400 141.570

26 124 T14 7 0.907 21.746 92.857 11.751 180.700 171.100

27 97 T15 3 1.213 25.692 96.429 11.609 182.180 167.370

28 114 T15 5 1.213 27.197 92.857 10.722 196.330 183.400

29 128 T15 7 1.213 24.096 100.00 10.254 171.960 155.030

30 83 T16 1 1.456 20.763 92.857 9.845 150.420 146.050

31 98 T16 3 1.456 19.843 100.00 8.880 162.270 141.960

32 103 T16 4 1.456 26.510 92.857 11.619 196.920 187.560

33 109 T16 5 1.456 21.950 100.00 10.003 178.000 166.320

34 120 T16 6 1.456 22.623 89.286 9.847 165.540 165.630

35 125 T16 7 1.456 23.783 100.00 10.895 181.920 177.450

;

**run;**

**proc print;**

**run;**

**Determination of parameters**

**data** ReportManoela; set ReportManoela;

ArginineIntake=(FeedIntake*(Level/100))*1000;

EggMass=(EggProduction/100)*EggWeight;

CPinEgg=(EggMass*(13/100)); /*/ 13% obtained from Mostafa et al. 2019 /*/

ArginEgg=CPinEgg*(5.41/100); /*/ 13% obtained from Mostafa et al. 2019 /*/

Deposition=ArginEgg*1000;

MBW=(((InitialBW+FinalBW)/2)/1000)**0.67;

FeedConversionRatio=(FeedIntake/EggMass);

FeedEfficiency=(EggMass/FeedIntake);

ChangeWG=(FinalBW-InitialBW);

CPBbodyComposition=(ChangeWG*(20.3/100))/56; /*/ 56 days Trial period /*/

ArginineChangeBW=(CPBBodyComposition*(9.40/100))*1000; /*/ 9.40=(10.76+8.04)/2 - Sharif et al 2019 /*/

EMMBW=EggMass/MBW;

y=Deposition;

y1=Deposition/MBW;

x=ArginineIntake/MBW;

Maintenance=(90*MBW);

x1=(ArginineIntake-Maintenance)/MBW;

x2=(ArginineIntake-(Maintenance+abs(ArginineChangeBW)))/MBW;

**run;**

**proc print; run;**

**Response table and ANOVA and significance for linear and quadratic effect**

**This procedure must be repeated for all variables**

**proc mixed data**=ReportManoela;

CLASS Level RP;

MODEL EggProduction = Level;

RANDOM RP/TYPE=AR(1) SUB=RP;

CONTRAST "EF.LIN" Level -3 -2 -1 0 1 2 3;

CONTRAST "EF.QUA" Level 5 0 -3 -4 -3 0 5;

**run**;

**Description of the maximum and minimum response for arginine deposition in the egg, maintenance requirement and total efficiency of arginine utilization**

Model adjusted - exponential

**proc nlmixed data**=ReportManoela;

**parms** Rmax=253 Rmin=7.4 k=0.0032 Xm=74;

MODEL y1~normal((Rmax-Rmin)*(1-exp(-k*(x-Xm)))+ Rmaxvar, errvar);

**random** Rmaxvar~normal(0, RPvar) subject=RP;

**predict** (Rmax-Rmin)*(1-exp(-k*(X-Xm)))out=ppp;

**ods** output dimensions=ddd;

**run**;

**data** ppp; set ppp;

**resid**=y1-pred;

**run**;

**proc means** noprint data=ppp; where y1 ne .;

var y1 resid;

**output** out=mmm uss=totss sserr css=ctotss rss1 n=nobs; run;

**data** ddd; set ddd; if descr='parameters'; run;

**data** mmm; merge mmm ddd;

rsquare =(ctotss-sserr)/ctotss;

adjrsquare = 1 -(sserr/(35-4-1))/(ctotss/35-1); run;

**proc print**; run;

**proc reg** data=ppp;

**model** resid=pred;

**run;**

**proc print;**

**run**;

**Model adjusted - saturation kinetics**

**proc nlmixed** data=ReportManoela;

**parms** Rmax=232 b=21.1 n=3.4 k=291;

model y1~normal(((b*k**n)+(Rmax*x**n))/(k**n+x**n)+Rmaxvar, errvar);

**random** Rmaxvar~normal(0, RPvar) subject=RP;

**predict** ((b*k**n)+(Rmax*x**n))/(k**n+x**n)+Rmaxvar out=ppp;

**ods** output dimensions=ddd;

**run**;

**data** ppp; set ppp;

**resid**=y1-pred;

**run**;

**proc means** noprint data=ppp; where y1 ne .;

var y1 resid;

**output** out=mmm uss=totss sserr css=ctotss rss1 n=nobs; run;

**data** ddd; set ddd; if descr='parameters'; run;

**data** mmm; merge mmm ddd;

rsquare =(ctotss-sserr)/ctotss;

adjrsquare = 1 -(sserr/(35-4-1))/(ctotss/35-1); run;

**proc print**;

**run**;

**proc reg** data=ppp;

model resid=pred;

**run;**

**proc print;**

**run**;

**Liquid efficiency of arginine utilization for egg production**

**Broken line using correction: Kt - total efficiency y1 versus x as a regressor**

**proc nlmixed** data=ReportManoela;

parms Rmax=250 U=12.698 R=230;

Z1=(x<R)*(R-x);

MODEL y1~normal (Rmax+U*(Z1) + Rmaxvar, errvar);

**random** Rmaxvar~normal(0, RPvar) subject=RP;

**predict** Rmax+U*(Z1) + Rmaxvar out=ppp;

**ods** output dimensions=ddd;

**run**;

**data** ppp; set ppp;

resid=y1-pred;

**run**;

**proc means** noprint data=ppp; where y1 ne .;

var y1 resid;

**output** out=mmm uss=totss sserr css=ctotss rss1 n=nobs; run;

**data** ddd; set ddd; if descr='parameters'; run;

**data** mmm; merge mmm ddd;

rsquare =(ctotss-sserr)/ctotss;

adjrsquare = 1 -(sserr/(35-3-1))/(ctotss/35-1); run;

**proc print; run**;

**proc reg** data=ppp;

model resid=pred;

**run;proc print;**

**run**;

**Broken line using correction: K liquid correcting maintenance, traditional y1 versus x1 as a regressor**

**proc nlmixed** data=ReportManoela;

parms Rmax=250 U=12.698 R=230;

Z1=(x1<R)*(R-x1);

MODEL y1~normal (Rmax+U*(Z1) + Rmaxvar, errvar);

**random** Rmaxvar~normal(0, RPvar) subject=RP;

**predict** Rmax+U*(Z1) + Rmaxvar out=ppp;

**ods** output dimensions=ddd;

**run**;

**data** ppp; set ppp;

resid=y1-pred;

**run**;

**proc means** noprint data=ppp; where y1 ne .;

var y1 resid;

output out=mmm uss=totss sserr css=ctotss rss1 n=nobs; run;

**data** ddd; set ddd; if descr='parameters'; run;

**data** mmm; merge mmm ddd;

rsquare =(ctotss-sserr)/ctotss;

adjrsquare = 1 -(sserr/(35-3-1))/(ctotss/35-1); run;

**proc print; run**;

**proc reg** data=ppp;

model resid=pred;

**run;proc print;**

**run**;

**Broken line using correction: K liquid correcting maintenance + mobilization y1 versus x2 as a regressor**

**proc nlmixed** data=ReportManoela;

parms Rmax=250 U=12.698 R=230;

Z1=(x2<R)*(R-x2);

MODEL y1~normal (Rmax+U*(Z1) + Rmaxvar, errvar);

**random** Rmaxvar~normal(0, RPvar) subject=RP;

**predict** Rmax+U*(Z1) + Rmaxvar out=ppp;

**ods** output dimensions=ddd;

**run**;

**data** ppp; set ppp;

resid=y1-pred;

**run**;

**proc means** noprint data=ppp; where y1 ne .;

var y1 resid;

**output** out=mmm uss=totss sserr css=ctotss rss1 n=nobs; run;

**data** ddd; set ddd; if descr='parameters'; run;

**data** mmm; merge mmm ddd;

rsquare =(ctotss-sserr)/ctotss;

adjrsquare = 1 -(sserr/(35-3-1))/(ctotss/35-1); run;

**proc print;**

**run**;

**proc reg** data=ppp;

model resid=pred;

**run;**

**proc print;**

**run**;
